# Relative fat mass at baseline and its early change may be a predictor of incident nonalcoholic fatty liver disease

**DOI:** 10.1038/s41598-020-74659-9

**Published:** 2020-10-15

**Authors:** Hwi Young Kim, Su Jung Baik, Hye Ah Lee, Byoung Kwon Lee, Hye Sun Lee, Tae Hun Kim, Kwon Yoo

**Affiliations:** 1 Department of Internal Medicine, College of Medicine, Ewha Womans University, 1071, Anyangcheon-ro, Yangcheon-gu, Seoul, 07985 Republic of Korea; 2 Healthcare Research Team, Health Promotion Center, Gangnam Severance Hospital, Seoul, Republic of Korea; 3 Clinical Trial Center, Ewha Womans University Medical Center, Seoul, Republic of Korea; 4 Division of Cardiology, Gangnam Severance Hospital Cardiovascular Center, Yonsei University Health System, Seoul, Republic of Korea; 5 Biostatistics Collaboration Unit, Medical Research Center, Yonsei University College of Medicine, Seoul, Republic of Korea

**Keywords:** Population screening, Liver

## Abstract

The relationship between changes in body components and the risk of nonalcoholic fatty liver disease (NAFLD) is not fully understood. We investigated the effects of body components and subsequent changes on incident NAFLD at follow-up ultrasound scanning in a longitudinal cohort. We included 9967 participants without NAFLD at baseline who underwent serial health examinations. Sex-specific, weight-adjusted skeletal muscle index (SMI__Wt_) was used. Mean follow-up duration was 48.5 ± 33.5 months. NAFLD developed in 2395 participants (24.0%). Body composition was measured using bioelectrical impedance analysis. The following baseline body components were significantly associated with incident NAFLD: the lowest and middle SMI__Wt_ tertiles in the normal-weight group (adjusted hazard ratio [aHR] = 2.20 and 1.54, respectively), and fat percentage in the normal-weight (aHR = 1.12), overweight (aHR = 1.05), and obese groups (aHR = 1.03) (all *P* < 0.05). Among 5,033 participants who underwent ≥ 3 health examinations, SMI__Wt_ increase between the first and second examinations was an independent protective factor against incident NAFLD in non-obese groups (*P* < 0.05). Increased fat percentage was an independent risk factor for incident NAFLD in all weight categories (*P* < 0.05). High fat mass at baseline may be a better predictor of incident NAFLD than muscle mass. Reciprocal changes in fat and muscle mass during the first year of follow-up predicted incident NAFLD in non-obese groups.

## Introduction

Nonalcoholic fatty liver disease (NAFLD) is one of the most common causes of chronic liver diseases, representing approximately 25% of global prevalence^1^. NAFLD is a spectrum of liver diseases related to metabolic abnormalities, ranging from simple hepatic steatosis to nonalcoholic steatohepatitis with varying degrees of fibrosis, or even cirrhosis^2^. Patients with NAFLD have increased risks of hepatic or non-hepatic malignancies, cardiovascular events, and increased mortality^3^.

Although obesity is a risk factor for NAFLD^4^, the relationship between body composition and NAFLD appears complex. Incident NAFLD development is associated with insulin resistance and weight gain^5^. However, body mass index (BMI) is an imperfect measurement of adiposity and metabolic abnormality that does not distinguish between fat and muscle. Cut-off values of BMI to determine obesity differ for ethnic groups^6^. Moreover, body composition cannot be readily predicted by BMI, given that similar BMI may represent variable degrees of visceral adiposity, which is known to predispose the development of NAFLD^7^.

Recent studies have shown that overweight and obesity in metabolically healthy individuals are associated with a greater risk of incident NAFLD than that in normal weight individuals^8^. Higher muscle mass showed an inverse correlation with incident NAFLD, and increase in muscle mass suggested beneficial effects in NAFLD development^9^. Notwithstanding, the complex relationship between weight change (or body composition) with time and NAFLD development has not been fully explored. In this study, we investigated the effects of baseline values and changes in body composition on the development of incident NAFLD in a longitudinal cohort.

## Results

### Baseline characteristics

**Figure 1:**
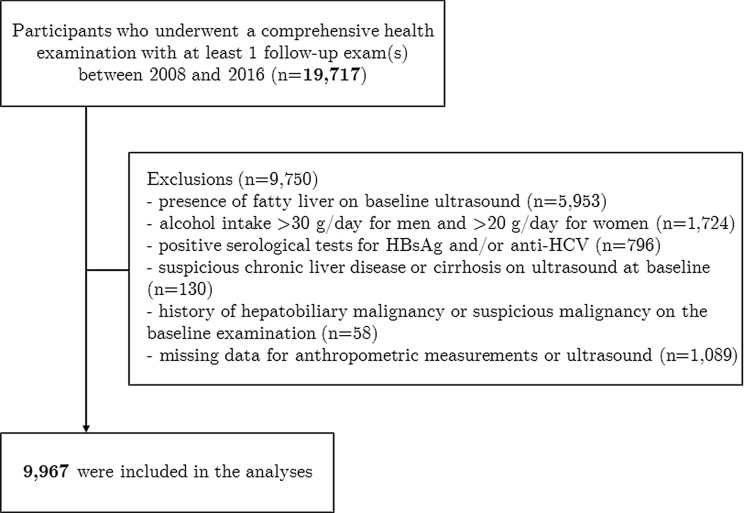
Flowchart for patient selection.

Overall, 9967 participants were included in the study (Fig. 1). The baseline characteristics of the study participants are summarized in Table 1. The mean age was 45.9 ± 10.8 years, and 6156 (61.8%) participants were men. The mean BMI was 22.3 ± 2.8 kg/m^2^. Study participants were categorized according to their BMI as underweight (n = 710, 7.1%), normal weight (n = 5457, 54.8%), overweight (n = 2182, 21.9%), and obese (n = 1618, 16.2%).

**Table 1:** Baseline characteristics of the study subjects.

	All (n = 9967)	Incident NAFLD (n = 2395)	No incident NAFLD (n = 7572)	P
Age (years)	45.9 ± 10.8	47.1 ± 10.1	45.6 ± 11.0	< 0.001
Sex, male (%)	3811 (38.2)	1321 (55.2)	2490 (32.9)	< 0.001
**BMI (kg/m**^**2**^**)**	22.3 ± 2.8	23.7 ± 2.7	21.8 ± 2.7	< 0.001
BMI < 18.5	710 (7.1)	47 (2.0)	663 (8.8)	
18.5 ≤ BMI < 23	5457 (54.8)	958 (40.0)	4499 (59.4)	
23 ≤ BMI < 25	2182 (21.9)	662 (27.6)	1520 (20.1)	
BMI ≥ 25	1618 (16.2)	728 (30.4)	890 (11.8)	
Waist–hip ratio	0.83 ± 0.08	0.87 ± 0.07	0.82 ± 0.08	< 0.001
Soft lean mass (kg)	42.0 ± 8.0	45.1 ± 8.4	41.0 ± 7.6	< 0.001
SMI__Wt_	69.7 ± 5.4	68.7 ± 5.5	70.0 ± 5.4	< 0.001
**Sex-specific SMI**_**_Wt**_** tertiles (%)**				
T1	3323 (33.3)	1183 (49.4)	2140 (28.3)	< 0.001
T2	3326 (33.4)	771 (32.2)	2555 (33.7)	
T3	3318 (33.3)	441 (18.4)	2877 (38.0)	
Fat mass (kg)	14.9 ± 4.4	16.7 ± 4.5	14.3 ± 4.2	< 0.001
Fat percentage (%)	24.6 ± 5.5	25.5 ± 5.5	24.3 ± 5.5	< 0.001
AST (IU/L)	20 (17–24)	20 (17–25)	19 (17–23)	< 0.001
ALT (IU/L)	16 (12–22)	19 (15–27)	15 (12–21)	< 0.001
GGT (IU/L)	17 (12–26)	21 (15–34)	15 (12–23)	< 0.001
Total cholesterol (mg/dL)	190.3 ± 34.3	194.1 ± 34.5	189.1 ± 34.1	< 0.001
Triglycerides (mg/dL)	86 (65–121)	109 (78–154)	81 (62–110)	< 0.001
LDL cholesterol (mg/dL)	119.3 ± 31.2	124.5 ± 31.6	117.7 ± 31.0	< 0.001
HDL cholesterol (mg/dL)	56.6 ± 13.1	52.1 ± 12.2	58.0 ± 13.0	< 0.001
Glucose (mg/dL)	91 (86–97)	94 (87–100)	91 (85–97)	< 0.001
Uric acid (mg/dL)	4.8 ± 1.3	5.2 ± 1.3	4.6 ± 1.2	< 0.001
WBC (10^3^/µL)	5.52 (4.66–6.57)	5.97 (5.04–7.11)	5.40 (4.56–6.41)	< 0.001
Hemoglobin (g/dL)	14.0 ± 1.6	14.5 ± 1.6	13.9 ± 1.5	< 0.001
Platelets (10^3^/µL)	260.7 ± 58.6	267.3 ± 57.7	258.5 ± 58.7	< 0.001
Creatinine (mg/dL)	0.8 (0.68–0.94)	0.9 (0.7–1.0)	0.8 (0.7–0.9)	< 0.001
eGFR (ml/min/1.73 m^2^)	89.6 ± 22.4	87.1 ± 21.4	90.4 ± 22.7	< 0.001
HSI	33.7 ± 3.4	34.0 ± 3.2	33.5 ± 3.5	< 0.001
TyG	8.3 ± 0.5	8.6 ± 0.5	8.3 ± 0.5	< 0.001
Hypertension, N (%)	1480 (14.8)	493 (20.6)	987 (13.0)	< 0.001
Diabetes, N (%)	263 (2.6)	107 (4.5)	156 (2.1)	< 0.001
SBP (mmHg)	118.8 ± 14.7	122.6 ± 14.5	117.6 ± 14.6	< 0.001
DBP (mmHg)	73.4 ± 9.9	76.1 ± 9.6	72.6 ± 9.8	< 0.001
Alcohol (≥ 1 time/week), N (%)	3,475 (50.9)	804 (55.1)	2,671 (49.7)	< 0.001
Current smoking, N (%)	904 (13.3)	298 (20.5)	606 (11.3)	< 0.001
Exercise (≥ 1 times/week), N (%)	4,056 (60.6)	896 (62.3)	3,160 (60.1)	0.137
**No. of components of metabolic syndrome, N (%)**				
0	6,150 (61.7)	1,124 (46.9)	5,026 (66.4)	< 0.001
1	2,696 (27.0)	785 (32.8)	1,911 (25.2)	
2	899 (9.0)	377 (15.7)	522 (6.9)	
≥ 3	222 (2.2)	109 (4.6)	113 (1.4)	

Frequencies and percentages for categorical variables, mean ± standard deviations for continuous variables.

*NAFLD* nonalcoholic fatty liver disease, *BMI* body mass index, *SMI*_*_Wt*_ weight-adjusted skeletal muscle index, *AST* aspartate aminotransferase, *ALT* alanine aminotransferase, *GGT* γ-glutamyl transpeptidase, *LDL* low-density lipoprotein, *HDL* high-density lipoprotein, *WBC* white blood cell, *eGFR* estimated glomerular filtration rate, *HSI* hepatic steatosis index, *TyG* triglyceride-glucose index, *SBP* systolic blood pressure, *DBP* diastolic blood pressure, *T1* lowest tertile, *T2* middle tertile, *T3* highest tertile.

During follow-up (mean follow-up duration, 48.5 ± 33.5 months), incident NAFLD was observed in 2395 participants (24.0%; incident rate 59.4 per 1000 person-years). Participants with incident NAFLD were more frequently older (47.1 vs 45.6 years), were men (55.2% vs 32.9%), had higher baseline BMI (23.7 vs 21.8), and were more likely to have metabolic abnormalities (53.1% vs 33.6%) than those without NAFLD (all *P* < 0.001). In the study groups, incident NAFLD developed in 6.6%, 17.6%, 30.3%, and 45.0% of the underweight, normal weight, overweight, and obese groups, respectively (*P* < 0.001) (Table 1). Calculation of serum fibrosis indices in participants with incident NAFLD demonstrated that an absolute majority was classified as low risk for all indices including NAFLD fibrosis score (< − 1.455: n = 2393, 99.9%), fibrosis score-4 (FIB-4, < 1.3: n = 1852, 77.3%), and aspartate aminotransferase-to-platelet ratio index (APRI, < 0.5: n = 2108, 88.8%).

### Relationships between baseline body composition parameters and incident NAFLD during follow-up among BMI subgroups

Given that baseline muscle and fat masses were significantly different among the subgroups according to BMI cut-off values (underweight, normal weight, overweight, and obese; Supplementary Table S1), associations among baseline body composition parameters and incident NAFLD were investigated in each BMI subgroup using multivariate Cox regression analysis (Table 2).

**Table 2:** Baseline risk factors for incident NAFLD according to BMI categories.

	BMI < 18.5			18.5 ≤ BMI < 23.0			23.0 ≤ BMI < 25.0			BMI ≥ 25.0		
	aHR	95% CI	*P*	aHR	95% CI	*P*	aHR	95% CI	*P*	aHR	95% CI	*P*
**Model 1**												
Lowest SMI__Wt_ tertile	NA			2.90	2.35–3.57	< 0.001	1.35	0.98–1.87	0.070	1.66	0.95–2.88	0.073
Middle SMI__Wt_ tertile	1.77	0.39–8.00	0.456	1.80	1.55–2.09	< 0.001	1.09	0.79–1.51	0.604	1.46	0.82–2.61	0.198
Fat percentage	1.07	0.96–1.18	0.223	1.16	1.13–1.18	< 0.001	1.07	1.03–1.11	< 0.001	1.05	1.02–1.07	< 0.001
**Model 2**												
Lowest SMI__Wt_ tertile	NA			2.20	1.77–2.73	< 0.001	1.22	0.88–1.70	0.235	1.46	0.84–2.54	0.185
Middle SMI__Wt_ tertile	2.11	0.45–9.99	0.345	1.54	1.33–1.79	< 0.001	1.01	0.73–1.40	0.972	1.40	0.78–2.50	0.260
Fat percentage	1.07	0.96–1.19	0.201	1.12	1.09–1.15	< 0.001	1.05	1.02–1.09	0.001	1.03	1.00–1.05	0.038

Model 1: adjusted for age, sex and smoking; Model 2: Model 1 and further adjusted for blood pressure, glucose, triglyceride, HDL and uric acid.

*BMI* body mass index, *aHR* adjusted hazard ratio, *CI* confidence interval, *SMI*_*_Wt*_ weight-adjusted skeletal muscle index, *HDL* high- density lipoprotein.

In the normal weight subgroup, participants in the lowest sex-specific, weight-adjusted skeletal muscle index (SMI__Wt_) tertile (T1) were significantly associated with an increased adjusted hazard ratio (aHR = 2.20; 95% confidence interval [CI], 1.77–2.73.; *P* < 0.001) for incident NAFLD after adjusting for age, sex, blood pressure, glucose, triglyceride, high-density lipoprotein (HDL), uric acid, and smoking status (Model 2, Table 2). Participants in T2 (the middle SMI__Wt_) were also significantly associated with an increased adjusted hazard ratio for NAFLD (aHR = 1.54; 95% CI 1.33–1.79; *P* < 0.001) (Model 2, Table 2).

In the other BMI subgroups (underweight, overweight, and obese), low muscle mass was not significantly associated with NAFLD development. On the contrary, fat percentage (FP) was significantly associated with incident NAFLD in the normal weight (aHR = 1.12; 95% CI 1.09–1.15; *P* < 0.001), overweight (aHR = 1.05; 95% CI 1.02–1.09; *P* < 0.001), and obese (aHR = 1.03; 95% CI 1.00–1.05; *P* = 0.04) subgroups.

**Figure 2:**
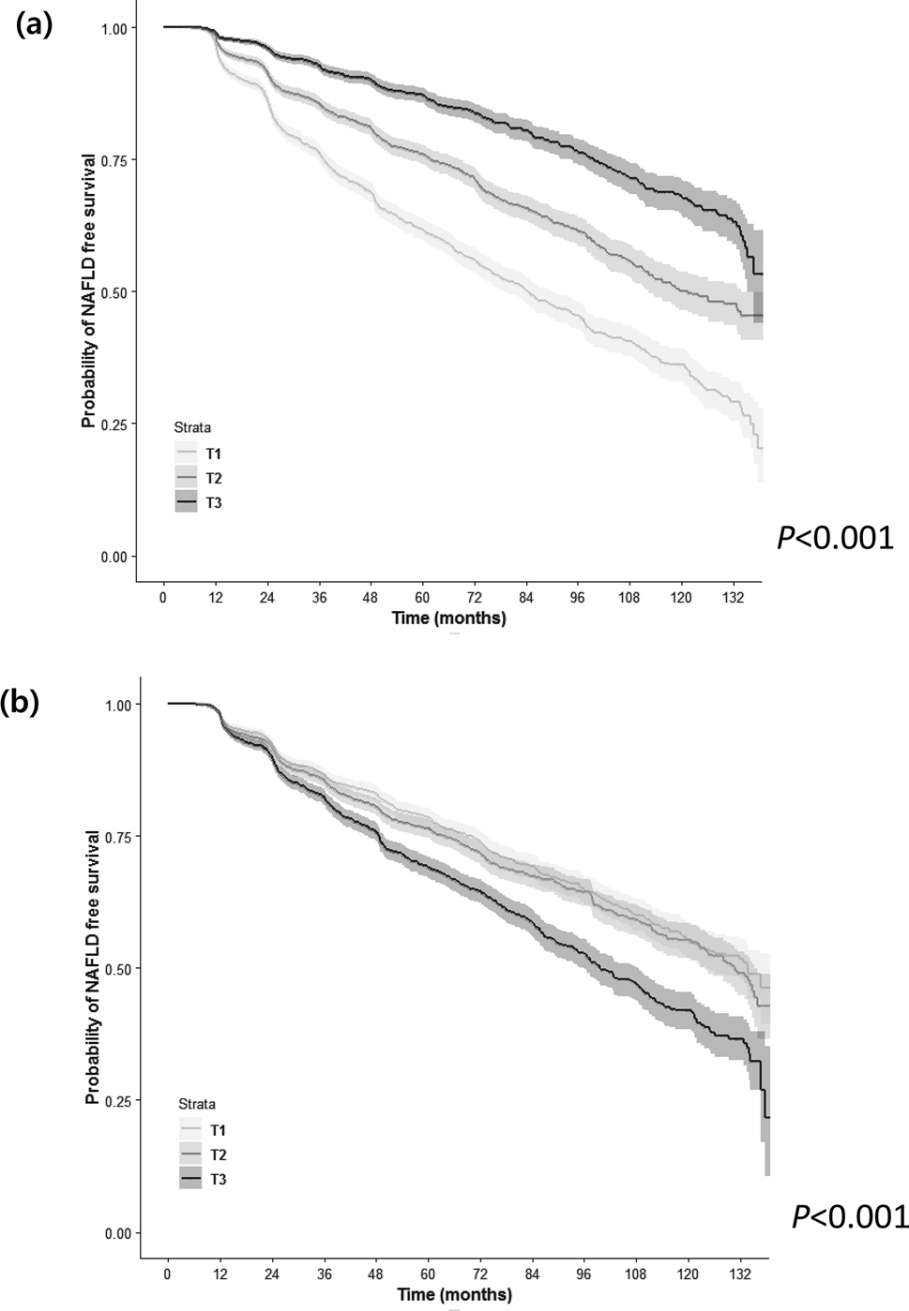
Kaplan–Meier curves for NAFLD-free survival according to weight-adjusted relative muscle and fat mass at baseline. (**a**) NAFLD-free survival according to sex-specific muscle mass tertiles. (**b**) NAFLD-free survival according to fat percentage tertiles. *NAFLD* nonalcoholic fatty liver disease, *T1* lowest tertile, *T2* middle tertile, *T3* highest tertile.

Figure 2 depicts the cumulative incidences of NAFLD according to SMI__Wt_ and FP tertiles. Participants in the lowest tertile of SMI__Wt_ (T1) showed the highest risk for incident NAFLD, followed by those in the middle and highest tertiles (Fig. 2a; *P* < 0.001 by log-rank test). Accordingly, participants in the highest tertile of FP had the highest risk for incident NAFLD, followed by those in the middle and lowest tertiles (Fig. 2b; *P* < 0.001 by log-rank test).

Among noninvasive fibrosis indices, NAFLD fibrosis score had a weak negative correlation with the baseline muscle mass and FIB-4 had a weak positive correlation with the baseline fat mass (Supplementary Table S2).

### Development of incident NAFLD and its relationship with changes in clinical parameters between first and second examinations

Of the entire study population, 5033 participants underwent a health examination three or more times during the study period, with a median interval of 1.2 years (interquartile range 1.0–2.0) between the first and second examinations. Changes in body composition and clinical parameters between the first and second examinations are summarized in Table 3. Participants in the underweight and normal-weight groups showed increases in body weight and FP, with a decrease in SMI__Wt_, whereas participants in the overweight and obese groups showed decreases in body weight, with no change or increase in SMI__Wt_ and no change or decrease in FP, respectively (all *P* < 0.001). In addition, significantly positive correlations were observed between increased weight, between the first and second examinations (ΔWt), and harmful changes in metabolic parameters (increased systolic and diastolic blood pressures, triglyceride, and glucose levels, and decreased HDL cholesterol; all *P* < 0.001) (Table 4). We further investigated the association between changes in these parameters and incident NAFLD development in the 5033 participants (Table 5). Increase in SMI__Wt_ between the first and second examinations was an independent protective factor against incident NAFLD in the underweight (aHR = 0.76; 95% CI 1.61–0.95; *P* = 0.015), normal-weight (aHR = 0.93; 95% CI 0.89–0.97; *P* = 0.002), and overweight groups (aHR = 0.90; 95% CI 1.84–0.96; *P* = 0.002) after adjustment for multiple covariates and baseline values. Moreover, FP increase was an independent risk factor for incident NAFLD in all weight categories (all *P* < 0.05).

**Table 3:** Changes in clinical parameters between baseline and follow-up examinations in subjects who underwent three or more health examinations (n = 5,033).

Changes in parameters	BMI < 18.5	18.5 ≤ BMI < 23.0	23.0 ≤ BMI < 25.0	BMI ≥ 25.0	P
ΔWeight (kg)	0.7 ± 1.5	0.3 ± 1.7	− 0.1 ± 2.1	− 0.5 ± 2.3	< 0.001
ΔSMI__Wt_	− 0.9 ± 2.1	− 0.4 ± 2.1	0.0 ± 2.2	0.3 ± 2.3	< 0.001
ΔFP (%)	0.8 ± 1.9	0.4 ± 1.8	0.0 ± 1.9	− 0.3 ± 2.0	< 0.001
ΔSBP (mmHg)	− 0.2 ± 8.1	− 0.2 ± 10.0	− 1.6 ± 10.7	− 1.6 ± 10.9	< 0.001
ΔDBP (mmHg)	− 0.1 ± 6.2	− 0.5 ± 6.5	− 1.0 ± 6.6	− 1.2 ± 6.7	0.012
ΔHDL (mg/dL)	0.2 ± 7.1	− 0.1 ± 6.5	0.0 ± 6.3	0.4 ± 5.9	0.230
ΔTG (mg/dL)	1.7 ± 26.5	3.4 ± 32.1	0.0 ± 39.6	− 2.5 ± 50.4	< 0.001
ΔGlucose (mg/dL)	1.3 ± 6.7	0.5 ± 7.9	0.9 ± 6.7	1.1 ± 10.7	0.081

*BMI* body mass index, *SMI*_*_Wt*_ weight-adjusted skeletal muscle index, *FP* fat percentage, *SBP* systolic blood pressure, *DBP* diastolic blood pressure, *HDL* high-density lipoprotein, *TG* triglyceride.

**Table 4:** Correlation between changes in metabolic parameters and changes in weight or body composition variables in subjects who underwent three or more health examinations.

(a) Correlation with weight change	ΔSMI__Wt_	ΔSBP	ΔDBP	ΔHDL	ΔTG	ΔGlucose	ΔFP
Correlation with ΔWeight (kg)	− 0.62	0.13	0.07	− 0.09	0.16	0.06	0.62
*P* for partial correlation	< 0.001	< 0.001	< 0.001	< 0.001	< 0.001	< 0.001	< 0.001

(b) Correlation with changes in muscle mass	ΔWt	ΔSBP	ΔDBP	ΔHDL	ΔTG	ΔGlucose	ΔFP
Correlation with ΔSMI__Wt_	− 0.62	− 0.07	− 0.07	− 0.02	− 0.08	− 0.06	− 0.94
*P* for partial correlation	< 0.001	< 0.001	< 0.001	0.265	< 0.001	< 0.001	< 0.001

*SMI*_*_Wt*_ weight-adjusted skeletal muscle index, *SBP* systolic blood pressure, *DBP* diastolic blood pressure, *HDL* high-density lipoprotein, *TG* triglyceride, *FP* fat percentage, *Wt* body weight.

**Table 5:** Changes in body composition parameters and risk of incident NAFLD according to BMI categories.

	BMI < 18.5			18.5 ≤ BMI < 23.0			23.0 ≤ BMI < 25.0			BMI ≥ 25.0		
	aHR	95% CI	*P*	aHR	95% CI	*P*	aHR	95% CI	*P*	aHR	95% CI	*P*
**Model 1**												
ΔSMI__Wt_	0.78	0.64–0.96	0.016	0.93	0.89–0.98	0.002	0.91	0.86–0.97	0.006	0.95	0.89–1.01	0.071
ΔFat percentage	1.48	1.19–1.86	0.001	1.10	1.05–1.16	< 0.001	1.16	1.07–1.25	< 0.001	1.10	1.03–1.18	0.005
**Model 2**												
ΔSMI__Wt_	0.76	0.61–0.95	0.015	0.93	0.89–0.97	0.002	0.90	0.84–0.96	0.002	0.95	0.89–1.01	0.094
ΔFat percentage	1.44	1.14–1.83	0.002	1.11	1.05–1.17	< 0.001	1.18	1.09–1.28	< 0.001	1.11	1.03–1.19	0.006

Model 1: adjusted for age, sex and baseline values; Model 2: Model 1 and further adjusted for changes in blood pressure, glucose, triglyceride, HDL and uric acid.

*BMI* body mass index, *aHR* adjusted hazard ratio, *CI* confidence interval, *SMI*_*_Wt*_ weight-adjusted skeletal muscle index, *HDL* high- density lipoprotein.

**Figure 3:**
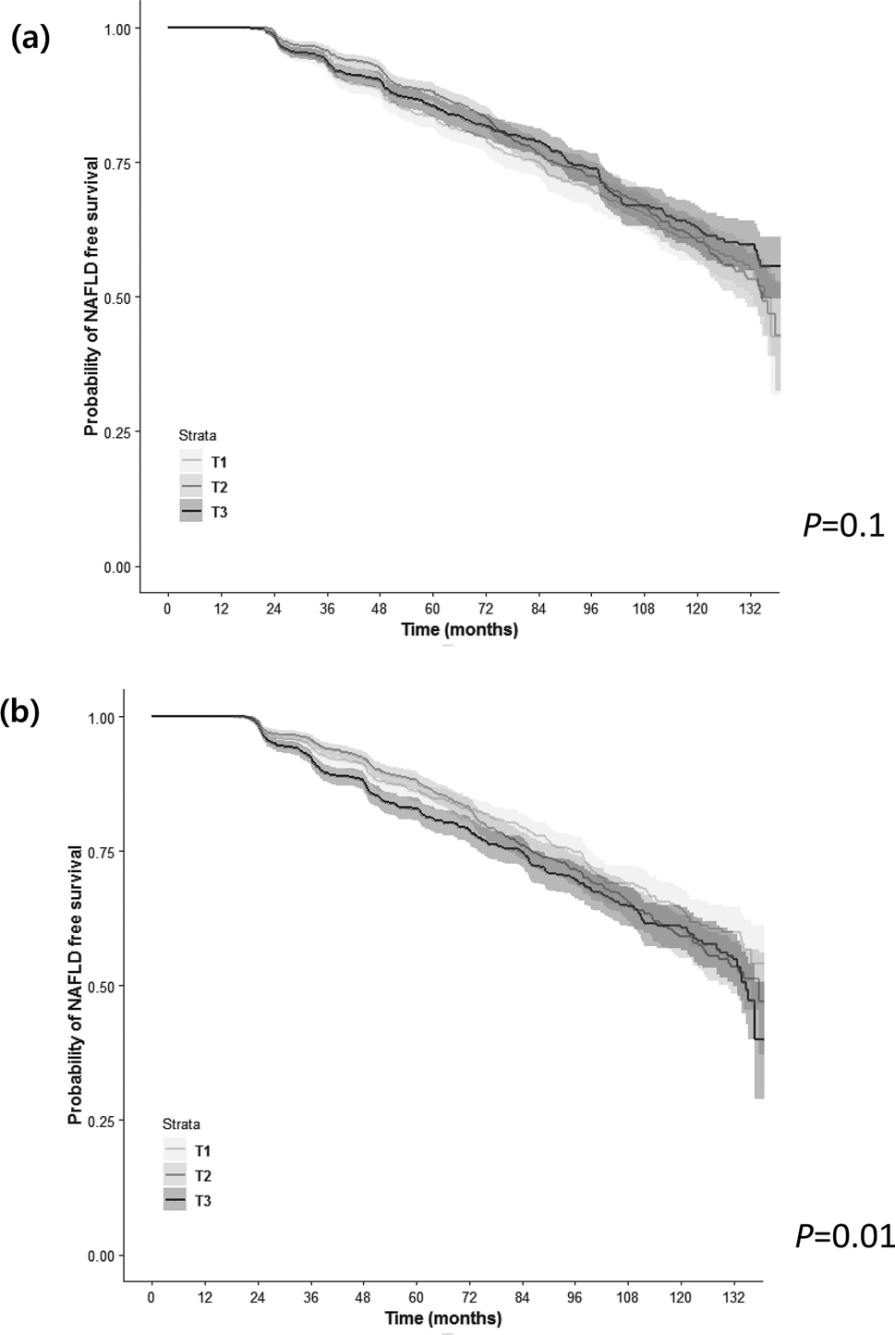
NAFLD-free survival duration according to changes in weight-adjusted relative muscle and fat mass during follow-up. (**a**) NAFLD-free survival according to tertiles of changes in sex-specific muscle mass. (**b**) NAFLD-free survival according to tertiles of changes in fat percentage. *NAFLD* nonalcoholic fatty liver disease, *T1* lowest tertile, *T2* middle tertile, *T3* highest tertile.

Figure 3 depicts the cumulative incidences of NAFLD according to the tertiles of changes in SMI__Wt_ and FP. Participants in the highest tertile of FP change showed the highest risk for incident NAFLD, followed by those in the middle and lowest tertiles (Fig. 3b; *P* = 0.01 by log-rank test). However, differences among the subgroups according to change in SMI__Wt_ tertiles were nonsignificant (Fig. 3a; *P* = 0.1 by log-rank test).

Assessment of the changes in body composition measurements showed no significant correlation with fibrosis indices (Supplementary Table S3).

### Post hoc analysis: metabolic dysfunction-associated fatty liver disease as the alternative outcome

According to the recently proposed diagnostic criteria^10^, 1914 subjects were identified as metabolic dysfunction-associated fatty liver disease (MAFLD) out of 2395 subjects with NAFLD (79.9%). Unlike the overweight and obese groups, the number of subjects was decreased 63.8% in the lean group (MAFLD, n = 17, vs NAFLD, n = 47) and 43.7% in the normal-weight group (MAFLD, n = 539, vs. NAFLD, n = 958). Associations between MAFLD development and low muscle mass/high FP at baseline were consistent with those on the development of NAFLD (aHR for MAFLD = 3.05 [95% CI 2.29–4.07] vs aHR for NAFLD = 2.20 [95% CI 1.77–2.73]) (Supplementary Table S4). However, in contrast to NAFLD, change in muscle mass was not significantly associated with the development of MAFLD in the low bodyweight group, and the effect was smaller in the normal- weight group (aHR for MAFLD = 0.86 [95% CI 0.81–0.91] vs aHR for NAFLD = 0.93 [95% CI 0.89–0.97]) (Supplementary Table S5).

## Discussion

This population-based longitudinal study suggested that high baseline fat mass rather than muscle mass may be a better predictor for incident NAFLD across BMI categories in participants without NAFLD at baseline. Moreover, reciprocal changes in fat and muscle masses during the first year of follow-up also predicted incident NAFLD in the non-obese population. Both findings were significantly associated with NAFLD development after adjustment for metabolic parameters.

The incidence rate of NAFLD in the present study (59.4 per 1000 person-years) was comparable to the pooled incidence rates in Asia (50.9 per 1000 person-years) in a recent systematic review^11^. The incremental incidence rates in subgroups with higher BMI categories reaffirmed the role of obesity in the development of NAFLD^12^. However, because BMI is an imperfect marker of adiposity or body fat distribution^13^, decreased or excess components of body composition at a given time and their changes with time were investigated to understand their roles in NAFLD development in various BMI categories. Low skeletal muscle mass has been suggested as a risk factor for NAFLD, considering the important role of skeletal muscles in insulin-mediated glucose disposal and secretion of myokines^14,15^.

Several recent studies have reported the relevance of sarcopenia as a risk factor for NAFLD, mostly in cross-sectional settings. According to a study based on a Korean nationwide survey, sarcopenia was independently associated with an increased prevalence of NAFLD defined by hepatic steatosis index score after adjusting for obesity or insulin resistance^16^. Other studies have also reported an association between low muscle mass and NAFLD prevalence in cross-sectional settings^17,18^. Recently, a longitudinal retrospective cohort study from Korea demonstrated that relative skeletal muscle mass at baseline was inversely associated with NAFLD development and positively associated with the resolution of NAFLD^9^. Because NAFLD incidence differed significantly among different BMI subgroups in the present study, we explored risk factors for incident NAFLD among the four different BMI subgroups. Consistent with former studies, the results of the present study for 9967 participants with at least two health examinations suggested associations between reduced relative muscle mass at baseline and incident NAFLD risk in the normal-weight group in a dose-dependent manner, after adjustment for multiple confounders.

Additionally, our results indicated that high baseline fat mass was a better predictor for incident NAFLD than low muscle mass in the normal-weight, overweight, and obese groups. From the Rotterdam study of a large population-based cohort, cross-sectional analysis showed that both high fat mass and low muscle mass were associated with NAFLD in normal-weight women (BMI < 25), whereas fat mass was a better predictor for NAFLD prevalence in both sexes^19^. Likewise, our results suggested that relative excess of fat mass predicted incident NAFLD better in all study populations except in participants with BMI < 18.5, whereas low muscle mass was only significant in the normal-weight subgroup. In addition, changes between the first and second health examinations were investigated to identify their relationship with the development of NAFLD in participants who underwent three or more health checkups during the study period (n = 5033). Even in the case of relatively small changes in body weight, muscle mass, and fat mass between the first and second examinations, differences among the four BMI subgroups were significant concerning the direction of changes (Table 3). Table 4 shows that changes in weight were correlated with changes in body composition and metabolic abnormalities, that is, even a small amount of weight gain was associated with blood pressure elevation, serum glucose and triglyceride increases, and HDL cholesterol decrease in addition to reduced muscle mass and increased fat mass. These findings suggest an evolving process toward metabolic abnormalities in NAFLD development as in previous studies, which reported insulin resistance or low adiponectin in non-obese participants with NAFLD^20,21^.

Considering the small, but significant, reciprocal changes in muscle and fat mass accompanying weight changes in our study participants, we further explored the relationship between changes in body composition parameters and incident NAFLD risk. A significant increase in incident NAFLD risk was noted per percent increase in fat mass after adjustment for baseline values and other confounders in all BMI categories between the first and second examinations with a median interval of 1.2 years (Table 5). However, the degree of increase in incident NAFLD risk per percent decrease in muscle mass showed less robustness overall, as well as a nonsignificant association in the obese group. A recent single-center retrospective study from Korea reported that a progressive increase in fat mass and loss of muscle mass with aging was significantly associated with incident NAFLD, particularly in non-obese participants, between baseline and follow-up health examinations at 10 years^22^. However, loss of muscle mass over a longer time period is an aging process^23^. Instead of evaluating the effect of aging on NAFLD development, we focused on the effect of early changes in body composition from the perspective of NAFLD prevention. Studies have demonstrated the efficacy of lifestyle intervention in preventing progression to diabetes in individuals with prediabetes, even compared with metformin^24,25^. Similarly, our results show that if participants with unfavorable body composition at baseline achieve an increase in muscle mass and more importantly decrease in fat mass over a 1- or 2-year period, even small amounts of such changes can reduce the risk of future NAFLD.

Generally, Asians have proportionately higher body fat for a given BMI than people of other races^26,27^. Non-obese Asian participants with NAFLD have a higher body fat content compared with those with comparable BMI without NAFLD^28^. Therefore, more robustness of fat mass compared with muscle mass in terms of NAFLD risk in the present study must be validated in other populations, including those in the West. Although there have been several Western studies on the relationship between body composition and NAFLD, which included advanced nonalcoholic steatohepatitis^29,30^, similar studies in a presumably healthy population are scarce, except for the Rotterdam study^19^. Despite its cross-sectional design, the results of the Rotterdam study at least underscore the relative importance of fat mass in NAFLD risk. Additionally, a recent randomized controlled trial from Hong Kong concluded that NAFLD remission was achieved with lesser weight reduction through lifestyle interventions in non-obese patients compared with obese patients^31^. The effect of fat mass and its changes on NAFLD development, particularly in the non-obese participants of the present study, suggest common pathophysiologic processes to those in the Hong Kong study in which non-obese patients achieved NAFLD remission with a modest degree of weight reduction^31^.

Recently, an international expert consensus statement recommended an updated definition of MAFLD instead of NAFLD^32^. MAFLD may more accurately reflects current knowledge of fatty liver diseases associated with metabolic dysfunction than NAFLD. When we applied MAFLD as the outcome, the change in muscle mass was not significantly associated with the development of MAFLD in the low bodyweight group and the effect was smaller in the normal-weight group. These results may have been affected by the smaller number of subjects in the lean group and normal-weight groups. However, the associations between MAFLD development and low muscle mass/high FP at baseline were consistent with the NAFLD results.

The present study had several limitations. First, NAFLD diagnosis was based on ultrasonography, instead of liver biopsy, which is the gold standard for the diagnosis of and severity assessment for NAFLD. Additionally, the use of ultrasonography for diagnosis raises concerns about possible misclassification bias of incident NAFLD diagnosis, which may weaken the associations found. However, recommending or performing liver biopsy in presumably healthy individuals could raise an ethical concern, considering that this study exclusively enrolled health checkup examinees. Second, the bioelectrical impedance analyzer used in this study was unable to determine the distribution (android or gynoid) of the fat mass, which is known to be related to metabolic abnormalities and NAFLD^19,33^. In addition, assessment of visceral adiposity, such as abdominal fat computed tomography, was unavailable. Third, the database lacked information on muscle function, such as grip strength, which is one of the various aspects in the assessment of sarcopenia^34^. Fourth, noninvasive techniques for NAFLD severity assessment, such as transient elastography, was not included in the health examination programs. Fifth, the present study included health checkup examinees from Korea, which may limit generalization of the results to other settings or ethnic groups with different cut-off values of BMI for obesity and different body composition patterns^27,35^. Lastly, we were unable to assess the effect of genetic variants due to the study design (lack of study samples). Recent genetic studies suggest a possible link between altered body composition and the development of NAFLD, for example, higher prevalence of patatin-like phospholipase domain-containing protein 3 (PNPLA3) [G] allele among non-obese individuals, an association between transmembrane 6 superfamily member 2 (TM6SF2) rs58542926 genotype and NAFLD that was independent of obesity, and interferon lambda 4 variant in non-obese nonalcoholic steatohepatitis^20,36,37^. More detailed studies are required to confirm the mechanistic link among genetic factors, body composition, and the risk of NAFLD.

In summary, although low muscle mass at baseline was a significant predictor for incident NAFLD in individuals with normal weight, high baseline fat mass predicted incident NAFLD in overweight and obese participants, as well as in those with normal weight, in this large-scale population-based study. In addition, reciprocal changes in fat and muscle masses during the first year of follow-up predicted incident NAFLD in participants with BMI < 25 kg/m^2^. The results of the present study add to the rationale of lifestyle interventions to prevent NAFLD development in individuals with high relative fat mass regardless of body habitus. Prospective validation is warranted for a bidirectional relationship between NAFLD and fat mass, that is, the effect of changes in body composition on incident NAFLD development, as well as resolution/progression of preexisting NAFLD with lifestyle intervention.

## Methods

### Study subjects and follow-up

In this study, 19,717 consecutive adult participants who had undergone a comprehensive health examination with two or more follow-up examinations between January 2007 and December 2018 at the Health Promotion Center of Gangnam Severance Hospital (Seoul, Korea) were included; some of the participants were offered health-screening examinations by their employers and the others voluntarily participated in health evaluation follow-up programs. The information obtained in the questionnaire included frequency of drinking alcohol per week and average amounts of alcohol consumed at a time. Alcohol intake was calculated based on drinking frequency and the amount of alcohol per drink for alcoholic beverages. The exclusion criteria were (1) fatty liver by baseline ultrasound (n = 5953), (2) alcohol intake > 30 g/day for men and > 20 g/day for women (n = 1,724), (3) positive serology for hepatitis B surface antigen and/or hepatitis C antibody (n = 796), (4) suspicious chronic liver disease (n = 130) or malignancy (n = 58) on baseline imaging studies, and (5) missing data on anthropometric measurements, body composition analysis findings, or ultrasound results (n = 1089). Finally, 9967 participants were included in the study (Fig. 1). Informed consent was obtained from all participants and only de-identified data were used from the routine health screening. The study protocol was approved by the Institutional Review Board of Gangnam Severance Hospital (IRB No. 3-2016-0280). The study protocol conformed to the ethical guidelines of the World Medical Association Declaration of Helsinki.

### Diagnosis of NAFLD

Abdominal ultrasonography was performed by seven experienced radiologists who were blinded to the clinical and laboratory characteristics of the study participants at the time of the examination. The diagnosis of fatty liver was based on the presence of at least two ultrasonographic features: (1) a diffuse increase in the fine echoes of the liver parenchyma compared with the spleen or kidney parenchyma; (2) ultrasound beam attenuation; and (3) poorly visualized intrahepatic structures^38^. Serum fibrosis markers were calculated for participants with incident NAFLD using laboratory values at the time of NAFLD diagnosis, including NAFLD fibrosis score, fibrosis score-4 (FIB-4), and aspartate aminotransferase-to-platelet ratio index (APRI)^39–41^.

The diagnosis of MAFLD was based on the evidence of hepatic steatosis (i.e., ultrasonographic fatty liver), in addition to one of the following three criteria: overweight/obesity (BMI ≥ 23 kg/m^2^), presence of type 2 diabetes mellitus, or evidence of metabolic dysregulation^10^.

### Clinical and laboratory assessments

A standardized, self-administered questionnaire was used to collect information on demographic characteristics, smoking status, preexisting medical conditions, and medication use. Height was measured to the nearest 0.1 cm using a stadiometer. Weight was measured to the nearest 0.1 kg and BMI was calculated as weight in kilograms divided by height in meters squared. The participants were categorized according to BMI based on the Korean Society for the Study of Obesity practice guidelines: underweight (BMI < 18.5), normal weight (18.5 ≤ BMI < 23), overweight (23 ≤ BMI < 25), and obese (BMI > 25)^42^. Blood samples were obtained following overnight fasting for 10–12 h. The laboratory tests included complete blood count, liver biochemistry, total cholesterol, triglycerides, high-density lipoprotein (HDL) cholesterol, glucose, hepatitis B surface antigen, and antibody to hepatitis C virus. Hypertension was defined as a systolic blood pressure ≥ 140 mmHg, diastolic blood pressure ≥ 90 mmHg, or current use of antihypertensives^43^. Diabetes was defined as a fasting serum glucose ≥ 126 mg/dL or self-reported insulin or antidiabetic use^44^.

### Measurement of body composition parameters

Body composition was measured using bioelectrical impedance analysis through tissue conductivity (X-SCAN Plus, Jawon Medical Co., Seoul, Korea)^45^, according to the manufacturer’s instructions. Briefly, study participants avoided eating or drinking 8 h before measurement and drinking alcohol 24 h before measurement. They grasped the handles of the device and contacted the electrodes while standing for up to 5 min. Skeletal muscle index was defined as total skeletal muscle mass/weight × 100, a modified formula according to the study of Janssen and colleagues^34,46–48^.

We divided participants using sex-specific, weight-adjusted SMI tertiles (SMI__Wt_; T1, T2, and T3). The relative proportion of body fat mass component was expressed as a percentage of total body weight^49^. Change in body composition between the first and second health examinations was calculated by subtracting baseline SMI__Wt_ or fat percentage (FP) from their corresponding values at the second health examination.

### Statistical analysis

Baseline characteristics are expressed as frequencies and percentages for categorical variables, and as mean ± standard deviations for continuous variables. Student's t-test, χ^2^ test, and analysis of variance were used to compare variables. Variables with skewed distribution were converted to natural logarithms for further analysis. The Kaplan–Meier method was used to describe the cumulative incidence of NAFLD at follow-up ultrasound scanning, and the log-rank test was used for comparison. Covariate variables were selected through a stepwise method of Cox proportional hazards analysis, excluding duplicate indicators among the significant variables related to NAFLD in univariate analysis, and were considered in multivariate analysis. Multivariate-adjusted Cox proportional hazards analysis was used to determine the hazard ratio of NAFLD at follow-up according to relevant variables. All tests were based on two-sided probability, and *P* < 0.05 was considered statistically significant. All analyses were performed using the SAS 9.4 (SAS Institute, Cary, NC, USA) and R 3.3.2 software packages (R Foundation for Statistical Computing, Vienna, Austria). R software (“survival” and “rms” packages) was used for the Kaplan–Meier analysis and survival plot.

## Supplementary information

Supplementary file1

## Data Availability

The data analyzed during the current study are available from the corresponding author on reasonable request.

## Abbreviations

NAFLD: Nonalcoholic fatty liver disease
SMI: Skeletal muscle index
BMI: Body mass index
aHR: Adjusted hazard ratio
FP: Fat percentage
FIB-4: Fibrosis score-4
APRI: Aspartate aminotransferase-to-platelet ratio index
HDL: High-density lipoprotein
T1: Lowest tertile
T2: Middle tertile
T3: Highest tertile
CI: Confidence interval
Wt: Body weight
MAFLD: Metabolic dysfunction-associated fatty liver disease
