# Supplementary material for: Relative fat mass at baseline and its early change may be a predictor of incident nonalcoholic fatty liver disease
Source: Sci Rep. 2020 Oct 15;10:17491. doi: 10.1038/s41598-020-74659-9 (PMC7567080; doi:10.1038/s41598-020-74659-9)
Supplement: Supplementary file 1 — Supplementary file1 [file 41598_2020_74659_MOESM1_ESM.docx]

**Relative Fat Mass at Baseline and Its Early Change May Be a Predictor of Incident Nonalcoholic Fatty Liver Disease**

**Authors**: Hwi Young Kim^1*^(M.D.), Su Jung Baik^2*^(M.D.), Hye Ah Lee^3^, Byoung Kwon Lee^2, 4^ (M.D.), Hye Sun Lee^5^, Tae Hun Kim^1^(M.D.), and Kwon Yoo^1^ (M.D.)

**Authors’ affiliations**: ^1^Department of Internal Medicine, College of Medicine, Ewha Womans University, Seoul, Republic of Korea; ^2^Healthcare Research Team, Health Promotion Center, Gangnam Severance Hospital, Seoul, Republic of Korea; ^3^Clinical Trial Center, Ewha Womans University Medical Center, Seoul, Republic of Korea; ^4^Division of Cardiology, Gangnam Severance Hospital Cardiovascular Center, Yonsei University Health System, Seoul; ^5^Biostatistics Collaboration Unit, Medical Research Center, Yonsei University College of Medicine, Seoul, Republic of Korea.

**Authors’ e-mails:** HYK, hwiyoung@ewha.ac.kr; SJB, splenic@yuhs.ac; HAL, khyeah @ewha.ac.kr; BKL, CARDIOBK@yuhs.ac; HSL, HSLEE1@yuhs.ac; THK, thkm@ewha.ac.kr; KY, yook57@ewha.ac.kr

* HYK and SJB contributed equally to this paper as co-first authors.

**Correspondence:** Hwi Young Kim, MD, PhD

Associate Professor, Department of Internal Medicine, College of Medicine, Ewha Womans University,1071, Anyangcheon-ro, Yangcheon-gu, Seoul 07985, Republic of Korea

Phone: +82-2-2650-5868, Fax: +82-2-2650-2837, Email: hwiyoung@ewha.ac.kr

**Running Title: Changes of body components and risk of NAFLD**

Supplementary Table S1. Baseline body composition parameters among subgroups according to body mass index.

| ` |  | BMI<18.5  (n = 710) | 18.5≤BMI<23  (n = 5,457) | 23≤BMI<25  (n = 2,182) | BMI≥25  (n = 1,618) | P |
| --- | --- | --- | --- | --- | --- | --- |
| Soft lean mass (kg) |  | 35.2 ± 4.4 | 39.7 ± 6.5 | 45.0 ± 7.5 | 48.7 ± 8.5 | <0.001 |
| SMI__Wt_ |  | 75.8 ± 4.2 | 70.7 ± 4.7 | 68.2 ± 4.9 | 65.4 ± 5.4 | <0.001 |
| Sex-specific SMI__Wt_ tertiles | T1 | 0 | 642 (11.8) | 1,301 (59.6) | 1,380 (85.3) | <0.001 |
|  | T2 | 10 (1.4) | 2,395 (43.9) | 724 (33.2) | 197 (12.2) |  |
|  | T3 | 700 (98.6) | 2,420 (44.4) | 157 (7.2) | 41 (2.5) |  |
| Fat mass (kg) |  | 8.5 ± 2.0 | 13.1 ± 2.5 | 16.8 ± 2.3 | 21.1 ± 3.8 | <0.001 |
| Fat percentage |  | 18.4 ± 4.2 | 23.6 ± 4.7 | 26.0 ± 4.9 | 28.9 ± 5.5 | <0.001 |
|  |  |  |  |  |  |  |
| Fat percentage tertiles | T1 | 620 (87.3) | 1,989 (36.5) | 560 (25.7) | 179 (11.1) | <0.001 |
|  | T2 | 89 (12.5) | 2,047 (37.5) | 657 (30.1) | 536 (33.1) |  |
|  | T3 | 1 (0.1) | 1421 (26.0) | 965 (44.2) | 903 (55.8) |  |

Abbreviations: BMI, body mass index; SMI__Wt_, weight-adjusted skeletal muscle index; T1, lowest tertile; T2, middle tertile; T3, highest tertile.

Supplementary Table S2. Correlation between body composition parameters and fibrosis indices in subjects with incident NAFLD.

|  | Correlation with SMI__Wt_ | *P* for partial correlation | Correlation with FP | *P* for partial correlation |
| --- | --- | --- | --- | --- |
| NAFLD fibrosis score | -0.137 | <0.001 | 0.137 | <0.001 |
| FIB-4 | -0.124 | <0.001 | 0.124 | <0.001 |
| APRI | 0.126 | <0.001 | -0.125 | <0.001 |

Abbreviations: SMI__Wt_, weight-adjusted skeletal muscle index; FP, fat percentage; NAFLD, nonalcoholic fatty liver disease; FIB-4, fibrosis score-4; APRI, aspartate aminotransferase-to-platelet ratio index

Correlation coefficients were obtained from the Pearson partial correlation after adjusting for body mass index.

Supplementary Table S3. Correlation between change of body composition parameters and fibrosis indices in subjects with incident NAFLD.

|  | Correlation with ΔSMI__Wt_ | *P* for partial correlation | Correlation with ΔFP | *P* for partial correlation |
| --- | --- | --- | --- | --- |
| NAFLD fibrosis score | 0.023 | 0.449 | -0.044 | 0.157 |
| FIB-4 | 0.038 | 0.223 | -0.059 | 0.062 |
| APRI | 0.0002 | 0.994 | -0.014 | 0.648 |

Abbreviations: SMI__Wt_, weight-adjusted skeletal muscle index; FP, fat percentage; NAFLD, nonalcoholic fatty liver disease; FIB-4, fibrosis score-4; APRI, aspartate aminotransferase-to-platelet ratio index

Correlation coefficients were obtained from the Pearson partial correlation after adjusting for body mass index and body composition values at baseline.

Supplementary Table S4. Baseline risk factors for incident MAFLD according to BMI categories.

|  | | BMI<18.5 | | | 18.5≤BMI<23.0 | | | | | 23.0≤BMI<25.0 | | | | BMI≥25.0 | | |
| --- | --- | --- | --- | --- | --- | --- | --- | --- | --- | --- | --- | --- | --- | --- | --- | --- |
|  | aHR | | 95% CI | *P* | | aHR | 95% CI | *P* | aHR | | 95% CI | *P* | aHR | | 95% CI | *P* |
| Model 1 |  | |  |  | |  |  |  |  | |  |  |  | |  |  |
| Lowest SMI__Wt_ tertile | NA | |  |  | | 4.53 | 3.44-5.96 | <0.001 | 1.42 | | 1.01-1.99 | 0.044 | 1.66 | | 0.95-2.88 | 0.073 |
| Middle SMI__Wt_ tertile | 1.49 | | 0.17-13.35 | 0.721 | | 2.26 | 1.85-2.77 | <0.001 | 1.14 | | 0.81-1.60 | 0.465 | 1.46 | | 0.82-2.61 | 0.198 |
| Fat percentage | 1.08 | | 0.92-1.29 | 0.350 | | 1.23 | 1.19-1.27 | <0.001 | 1.07 | | 1.03-1.11 | <0.001 | 1.05 | | 1.02-1.07 | <0.001 |
| Model 2 |  | |  |  | |  |  |  |  | |  |  |  | |  |  |
| Lowest SMI__Wt_ tertile | NA | |  |  | | 3.05 | 2.29-4.07 | <0.001 | 1.28 | | 0.91-1.81 | 0.157 | 1.46 | | 0.84-2.54 | 0.185 |
| Middle SMI__Wt_ tertile | 2.21 | | 0.20-24.09 | 0.515 | | 1.81 | 1.47-2.22 | <0.001 | 1.05 | | 0.75-1.48 | 0.767 | 1.40 | | 0.78-2.50 | 0.260 |
| Fat percentage | 1.07 | | 0.89-1.28 | 0.480 | | 1.17 | 1.13-1.21 | <0.001 | 1.06 | | 1.02-1.10 | 0.003 | 1.03 | | 1.00-1.05 | 0.038 |

Model 1: adjusted for age, sex and smoking; Model 2: Model 1 and further adjusted for blood pressure, glucose, triglyceride, HDL and uric acid.

Abbreviations: BMI, body mass index; aHR, adjusted hazard ratio; CI, confidence interval; SMI__Wt_, weight-adjusted skeletal muscle index; HDL, high- density lipoprotein.

Supplementary Table S5. Changes in body composition parameters and risk of incident MAFLD according to BMI categories.

|  | BMI<18.5 | | | 18.5≤BMI<23.0 | | | 23.0≤BMI<25.0 | | | BMI≥25.0 | | |
| --- | --- | --- | --- | --- | --- | --- | --- | --- | --- | --- | --- | --- |
|  | aHR | 95% CI | *P* | aHR | 95% CI | *P* | aHR | 95% CI | *P* | aHR | 95% CI | *P* |
| Model 1 |  |  |  |  |  |  |  |  |  |  |  |  |
| ΔSMI__Wt_ | 0.77 | 0.56-1.05 | 0.096 | 0.86 | 0.81-0.92 | <0.001 | 0.89 | 0.83-0.95 | <0.001 | 0.95 | 0.89-1.01 | 0.071 |
| ΔFP | 1.71 | 1.19-2.45 | 0.004 | 1.21 | 1.13-1.30 | <0.001 | 1.19 | 1.10-1.29 | <0.001 | 1.11 | 1.03-1.19 | 0.005 |
| Model 2 |  |  |  |  |  |  |  |  |  |  |  |  |
| ΔSMI__Wt_ | 0.75 | 0.49-1.15 | 0.190 | 0.86 | 0.81-0.91 | <0.001 | 0.88 | 0.82-0.94 | <0.001 | 0.95 | 0.89-1.01 | 0.094 |
| ΔFP | 1.60 | 1.01-2.53 | 0.047 | 1.23 | 1.14-1.32 | <0.001 | 1.22 | 1.12-1.32 | <0.001 | 1.11 | 1.03-1.19 | 0.006 |

Model 1: adjusted for age, sex and baseline values; Model 2: Model 1 and further adjusted for changes in blood pressure, glucose, triglyceride, HDL and uric acid.

Abbreviations: BMI, body mass index; aHR, adjusted hazard ratio; CI, confidence interval; SMI__Wt_, weight-adjusted skeletal muscle index; FP, fat percentage; HDL, high-density lipoprotein
